# Evolutionary Analyses of Sequence and Structure Space Unravel the Structural Facets of SOD1

**DOI:** 10.3390/biom9120826

**Published:** 2019-12-04

**Authors:** Sourav Chowdhury, Dwipanjan Sanyal, Sagnik Sen, Vladimir N. Uversky, Ujjwal Maulik, Krishnananda Chattopadhyay

**Affiliations:** 1 Protein Folding and Dynamics Group, Structural Biology and Bio-informatics Division, CSIR-Indian Institute of Chemical Biology, 4 Raja S.C.Mullick Road, Kolkata 700032, India; sourav_chowdhury@fas.harvard.edu (S.C.); dwipanjan92@gmail.com (D.S.); 2 Chemistry and Chemical Biology, Harvard University, 12 Oxford Street, Cambridge, MA 02138, USA; 3 Department of Computer Science, Jadavpur University, Kolkata 700032, India; sagnik.sen2008@gmail.com (S.S.); umaulik@cse.jdvu.ac.in (U.M.); 4 Department of Molecular Medicine and USF Health Byrd Alzheimer’s Research Institute, Morsani College of Medicine, University of South Florida, 12901 Bruce B. Downs Blvd. MDC07, Tampa, FL 33612, USA; vuversky@health.usf.edu; 5 Laboratory of New Methods in Biology, Institute for Biological Instrumentation, Russian Academy of Sciences, Pushchino 142290, Moscow Region, Russia

**Keywords:** superoxide dismutase, sequence space analysis, mutual information, direct information, structure network analysis, betweenness centrality

## Abstract

Superoxide dismutase (SOD) is the primary enzyme of the cellular antioxidant defense cascade. Misfolding, concomitant oligomerization, and higher order aggregation of human cytosolic SOD are linked to amyotrophic lateral sclerosis (ALS). Although, with two metal ion cofactors SOD1 is extremely robust, the de-metallated apo form is intrinsically disordered. Since the rise of oxygen-based metabolism and antioxidant defense systems are evolutionary coupled, SOD is an interesting protein with a deep evolutionary history. We deployed statistical analysis of sequence space to decode evolutionarily co-varying residues in this protein. These were validated by applying graph theoretical modelling to understand the impact of the presence of metal ion co-factors in dictating the disordered (apo) to hidden disordered (wild-type SOD1) transition. Contact maps were generated for different variants, and the selected significant residues were mapped on separate structure networks. Sequence space analysis coupled with structure networks helped us to map the evolutionarily coupled co-varying patches in the SOD1 and its metal-depleted variants. In addition, using structure network analysis, the residues with a major impact on the internal dynamics of the protein structure were investigated. Our results reveal that the bulk of these evolutionarily co-varying residues are localized in the loop regions and positioned differentially depending upon the metal residence and concomitant steric restrictions of the loops.

## 1. Introduction

Protein molecules are nature’s architectural marvels. They are crafted to perform a defined function which gives them their biological relevance [1]. In this study, we unraveled the evolutionary fingerprints in the structure of superoxide dismutase (SOD) with the aim to explore how the residues that are significant from an evolutionary perspective influence the structural integration of the protein. Superoxide dismutase is of immense importance from an evolutionary standpoint, since it had its origin at a significant geo-biological transition, the Great Oxidation Era. This geological era witnessed the rise and dominance of cyano-bacterial life forms which, in turn, was responsible for enriching the Earth’s atmosphere with molecular oxygen. The inundation of Earth’s atmosphere with molecular oxygen was coupled with the rise of oxygen-based metabolism and, hence, oxygen-based life forms [2]. Molecular oxygen is extremely prone to erroneous reduction, resulting in the generation of superoxide free radicals. These free radicals are reactive and highly toxic to the cellular machinery and membrane integrity [3,4,5]. Cells had to evolve ways to counteract the toxic impact of oxygen which led to the evolution of a highly orchestrated network of antioxidants in a defense system, where SOD is the primary antioxidant [2]. Therefore, the evolution of oxygen-based life is integrally coupled with the co-evolution of an antioxidant defense system, and, more importantly, evolution of SOD [2]. The rise of atmospheric oxygen was a critical event in biological evolution [5]. Some crucial evolutionary events, such as the birth of eukaryotes and the explosion of animal diversity in the Cambrian era, have been linked to elevated atmospheric oxygen [6]. Currently, solutions to questions related to planet oxygenation depend largely on geochemical methodologies [7].

Superoxide dismutase is found to exist in various canonical isomeric states [5,8]. The isomers differ in terms of the metal ion co-factors, although other functions remain identical. Human cells are armed with two canonical isoforms of SOD [5,8]. The mitochondrial SOD has Mn as the co-factor, and its rise and evolution can be traced back to bacterial endosymbiosis [8,9]. Cytosolic SOD, on the contrary, has Cu and Zn as the metal ion cofactors, and, hence, termed as Cu–Zn SOD or SOD1 [8,10]. This metalloenzyme exists as a homodimer, where each monomer is composed of a β-barrel and seven loops. Out of these loops, loop IV and VII are extremely significant from structural and functional perspectives. Six out of the seven metal co-ordination sites are positioned into the aforementioned two loops. A redox-active Cu ion is attached to the protein by its interaction with the four histidine residues: His 46, His 48, His 63, and His 120. A Zn co-ordination pocket is formed by the interaction between Zn ion and residues His 63, His 71, His 80, and Asp 83 [11]. Out of these seven metal binding residues, His 120 is located in the loop VII, His 48 is in the β-strand, and the remaining five residues are all positioned in loop IV. Importantly, these loop regions have been directly implicated in the misfolding and subsequent cytotoxic aggregation of SOD1, which lead to the fatal neurodegenerative implications and a diseased state termed amyotrophic lateral sclerosis (ALS) [11,12,13]. More than 100 different mutations have been associated with ALS [12,13]. Furthermore, the elucidation of combined sequential events of zinc acquisition, chaperone-mediated copper loading pathways, and functional activation through dimerization of SOD is integrated with the orchestration of loop regions [14,15,16]. Therefore, a comprehensive study of these two loop regions would offer better insights into the internal dynamics of this protein and the relevance of these loops to SOD1 physiology and function.

Several recent studies have been performed to understand the residue patch of SOD1 responsible for ALS. Probable aggregate structures of those disease mutants and their impact were also studied in cells [17,18]. But there is a lack of information on the effect of evolutionarily important residues on the emphasized disease mechanism. In this study, we unraveled the evolutionary fingerprints in the structure of SOD with the aim to explore how the regions that are significant from an evolutionary perspective influence the structural integrity of the protein. The evolutionary analysis involved understanding of the co-variation of amino acid residues in terms of mutual information (MI) and direct information (DI) by deploying direct coupling analysis (DCA) on SOD1 sequence space, comprising almost 4000 sequences from the SOD_Cu (PF00080) family. Our evolutionary coupling analysis identified the local regions of SOD1, where the bulk of the coupled pairs are lodged, reflecting the importance of discrete sub-structural areas in SOD1, which are under strong evolutionary selection.

As evidenced by the PONDR^®^-based disorder analysis, the loop regions (loop IV and VII) of the metal-depleted apo-SOD1 had two stretches of extended disorder, intrinsically disordered domain I and II (IDD I: residue 49–82 and IDD II: residue 121–142) [19]. On the contrary, the metalized wild-type SOD1 (WT SOD1) is very robust and possesses high thermal and chemical stability [13]. Therefore, the metal co-ordination of SOD1 is integral to this protein’s transition from the low stability disordered state (which is crucial for interaction with metal ions) to very stable ordered state required for subsequent biological functions of this protein. We resorted to structure–network analysis to understand how the inner organization of the pair-wise linked amino acids change, impacting the internal dynamics of SOD1 as this protein transcends from its apo-state to metallated form. For this purpose, we used a number of protein variants which either have a disrupted metal binding site or a mutated loop stretch. Stretch mutants were generated by means of mutations that were reported to be involved in ALS (retrieved from UniProt). These mutations were reported to promote aggregate formation by different distinct mechanisms. Some missense mutations were reported to distort the Zn binding [20] and some to decrease the metal ion coordination affinities that lead to the formation of aggregates [13]. Some of the selected residues were reported to reduce the net charge of the protein molecule at pH 7.4 [13]. We also used the fully metallated WT protein and completely metal-free apo-protein as two extreme controls. The variants selected for our study stand important as the metal micro-environment and the loop flexibility is intrinsically coupled.

Although there are multiple reports reflecting upon the importance of the electrostatic loops [21,22,23,24] in SOD1 and the role of metal ions in terms of crafting the structural integrity in SOD1, there is a lack of understanding as to how these residues are interdependent and whether they are under evolutionary selection pressure. Deploying co-evolution analysis on a wide array of sequences, we have been able to retrieve residues which are co-evolving and, hence, evolutionarily coupled.

Our collective inferences drawn from sequence space and structural analysis suggest the critical importance of loop regions along with the residue specific contribution in deciding the global conformational fate of SOD1 and the concomitant transition from disorder to order upon sequential metal co-ordination.

## 2. Materials and Methods

In this section, the proposed framework for complete multi-level screening is discussed. We started with the sequence screening of SOD1 from Pfam. Following that, we performed multiple sequence alignment (MSA) and sequence space analysis. The structure space analysis was started by generating in-silico computational models of structural mutants. Subsequently, we built networks for each protein mutant types. The process is elaborately discussed below.

### 2.1. Sequence Space Analysis

#### 2.1.1. Data Pre-Processing

For the sequence-based analysis, we started with the superoxide dismutase family, which has almost 4000 sequences from 1893 species from Pfam. A hidden Markov model (HMM)-based MSA was performed and the output was stored as MS_aligned_. A two-fold computational method was applied on MS_aligned_ sequentially. The detailed method is described below.

#### 2.1.2. Computational Analysis of the Intrinsic Disorder Predisposition of Human SOD1

The peculiarities of intrinsic disorder distribution within the amino acid sequence of human SOD1 (UniProt ID: P00441) were analyzed by a set of commonly used per-residue disorder predictors, such as PONDR^®^ VLXT [25], PONDR^®^ VSL2 [26], PONDR^®^ VL3 [26], PONDR^®^ FIT [27], and two forms of IUPred suitable for prediction of short and long intrinsically disordered regions, IUPred_short and IUPred_long, respectively [28,29]. We also analyzed the mean disorder propensity for these proteins by averaging the disorder profiles of individual predictors. The use of consensus for evaluation of intrinsic disorder was based on empirical observations showing that such an approach usually increases the predictive performance compared to the use of a single predictor [30,31,32]. In these analyses, predicted disorder scores above 0.5 were considered to correspond to the disordered residues and regions.

#### 2.1.3. Coupling Study

Throughout evolution, slow changes are observed in protein sequences while the fold of native structures remains unaffected [33]. Amino acid residues that do not change throughout evolution are considered conserved residues. They introduce significant influences on maintaining the protein structure and function. Mutations in non-conserved regions may also lead to structural disruption and functional disorders. The impact of change in shape, size or other physico-chemical properties by mutation at one position must be nullified or counterbalanced by compensatory alteration in another residue in close proximity to the 3D folded structure [34], i.e., co-variation of two residues in terms of evolution is extremely significant in order to preserve or restore the structure and activity of protein [35].

To understand the dependence of one position on another (i.e., positional correlation or coevolution propensity of amino acids along the sequence), mutual information (MI) theory was implemented. More precisely, using MI, the coupling propensities among two residues were calculated considering the effects of whole sequence space. Mutual information is a type of mathematical formulation, which was applied to establish the relationship between two random positions in an amino acid sequence. In Equation (1), a generic form of the MI calculation is given, where *P*(*x*,*y*) is considered as a joint probability, and *P*(*x*) and *P*(*y*) are individual probability. The resultant MI scores represent the coupling strength among two amino acid positions/residues (*MI_Cij_*). Here, *P*(*x*,*y*), *P*(*x*), and *P*(*y*) are replaced with specific functions; i.e., *P*(*A*,*B*), *P*(*A*), and *P*(*B*) (Equation (4)), respectively.

(1) $$MI=\underset{y\epsilon Y}{\sum }\underset{x\epsilon X}{\sum }P\left(x,y\right)\log(\frac{P\left(x,y\right)}{P\left(x\right)P\left(y\right)})$$

(2) $$P_{i^{\left(A\right)}}=\frac{1}{\lambda +M_{eff}}\left(\frac{\lambda }{q}+\overset{M}{\underset{a=1}{\sum }}\frac{1}{m^{a}}\delta_{A,A_{i}^{a}}\right)$$

(3) $$P_{ij^{\left(A,B\right)}}=\frac{1}{\lambda +M_{eff}}\left(\frac{\lambda }{q^{2}}+\overset{M}{\underset{a=1}{\sum }}\frac{1}{m^{a}}\delta_{A,A_{i}^{a}}\delta_{B,A_{i}^{a}}\right)$$

*P_i_*(*A*) represents the singular site frequency, probability of finding amino acid type *A* at the *i*th position in the sequence (Equation (2)), *λ* is a random parameter, and *M_eff_* is given as a total weight over all sequences where *M* numbers of MSA rows are given based on distribution of *q* alphabets (20 amino acids, 1 gap) over each sequence length *L*. Likewise, *m^a^* represents sequence identity scoring and *δ* indicates the Kronecker’s symbol. *P_j_*(*B*) (for amino acid type *B* at *j*th position) is equivalent to *P_i_*(*A*). *P*_*i*,*j*_(*A*,*B*) represents joint probability of observing amino acid type *A* at position *i* and amino acid type *B* at *j*th position in the amino acid sequence [33] (Equation (3)).

Secondary correlation between non-interacting residues may arise from correlations among substitution patterns of the interacting ones [35]. In order to investigate native contacts in a more specific way, direct couplings were needed to be understood explicitly. A major shortcoming of the covariance study; i.e., the MI theory, is that it cannot disentangle direct correlations from indirect ones. Therefore, the direct-coupling analysis (DCA) [35] was employed, which produces direct information (DI; Equation (4)) that measures how directly coupled two sites are in the MSA.

(4) $$DI_{ij}=\underset{AB}{\sum }P_{ij}^{\left(dir\right)}\left(A,B\right)\;ln\frac{P_{ij}^{\left(dir\right)}\;\left(A,B\right)}{P_{i}\left(A\right)P_{j}\left(B\right)}$$

Here $P_{ij}^{\left(dir\right)}$ represents reweighted frequency counts to introduce two residues for DI. The coupling propensity of two residues depends on their coupling strength. Therefore, we selected some coupled pairs corresponding to top MI and DI values. To get an idea about the residue pairs that were more likely to be coupled (i.e., to be co-varied evolutionary), only those residue pairs imposing contacts were considered.

#### 2.1.4. Graph Theoretical Modelling

From MI_Cij_ and DI_Cij_, two bigraphs were designed individually (as each of them had two disjoint set of coupling pairs). These were represented as two bigraph-based weighted networks, i.e., G_MI_ and G_DI_ for MI_Cij_ and DI_Cij_, respectively. (V_MI_, E_MI_) ∈ G_MI_|V_MI_ represented residues and E_MI_ symbolized weighted edges among coupled pairs, considering MI scores as weights. Similarly, (V_DI_, E_DI_) ∈ G_DI_|V_DI_ represented residues and E_DI_ denoted weighted edges between directly correlated coupled pairs, considering DI scores as weights. Based on weighted vertices (potential coupling strength) from the networks, these two networks were split into multiple communities with higher modularity (dense networks or strongly connected networks with a maximum number of nodes carried higher modularity) depending upon edge betweenness (number of total shortest paths passing through the edge) scoring.

In order to understand which residues were extremely critical in terms of their contribution towards the global stability of the structure, maximal clique from each of the networks was derived. Clique is a complete sub-graph, whereas maximal clique is a special type of complete subgraph that cannot be extended by including one more adjacent vertex. Maximal clique revealed the highly connected regions with a maximum number of nodes (residues).

### 2.2. Structure Network Analysis

#### 2.2.1. Model Building

The WT model was resorted from PDB (PDB ID: 2XJK) [36]. Three variants (de-metallated, apo SOD1, and mono-metallated, Cu-SOD and Zn-SOD forms) of the protein were generated by performing point mutation at the metal co-ordination site using I-TASSER [37]. Here, instead of selecting the available crystal structures of the apo form, we substituted the histidine 63 residue by phenylalanine and built the abovementioned mutant. This apo mutant was performed by single site mutation (H63F). Similarly, in the case of the generation of the Zn-SOD1 variant, we substituted histidine 120 by phenylalanine (H120F) and for Cu-SOD1, histidine 71 was mutated by phenylalanine residue (H71F). Again, stretch mutants (L4, L7, L4S SOD1) were generated by referring to sequences (retrieved from UniProt), which have been reported to have implications in protein aggregation leading to ALS [13] by different discrete mechanisms as mentioned earlier. Both L4 and L7 stretch mutants were generated by substituting ALS-involved residues in loop IV and loop VII, respectively (Table 1). The L4S variant involved stretch mutations on both the loop IV and the β-strand (Table 1). These models were further subjected to structure network analysis.

#### 2.2.2. Structure Network Analysis

Analysis and prediction of dynamics associated with complex systems can be explained and represented using network concepts. In general, a complex system is composed of elements interacting with one another (nodes and vertices) bound together by links like contacts, edges, and interactions. In graph-based networks, links represent interactions among two pairs of elements. Weight associated with a link characterizes the strength of interaction. Overlapping modules can, in turn, be dissected from the network (i.e., communities, groups) which often form a hierarchical arrangement.

A structure network’s representation of a protein is a measure of the topology of complex 3D structure irrespective of the secondary structure and folding type [38,39]. In this approach, a weighted graph G was constructed that represents a 3D PDB structure, (V, E) ∈ G, where *V* (*V* = *V*_1_, *V*_2_ … *V_n_*) represents residues as nodes and E (E = E_1_, E_2_, … E_n_) designates edges representing pairwise interaction. The internal motions and intrinsic dynamics of proteins dictate the global protein structure and, hence, the function and activity. We used a normal mode analysis (NMA) for predicting the functional motions in SOD1 [40]. We resorted to an elastic network model NMA using C-alpha force field. Followed by NMA, a correlation analysis was performed to identify protein segments with correlated motions, and a cross-correlation matrix was generated. By means of correlation network analysis, a full residue network of different mutant models of Cu–Zn SOD1 along with the WT protein were generated. These networks were split into a highly correlated coarse-grained community cluster network using the Girvan–Newman clustering method, where the highly interacting residues were grouped together in clusters [41].

The role of a particular node as a connector among other nodes, (i.e., the importance of a residue to a network in its functioning as a bridging point) is represented by measuring the number of shortest paths passing through that particular node [42]. Betweenness centrality characterizes the regions of a protein that show differences in coupled motions derived from different mutants as well as the WT protein. Residues having a significant contribution to the intrinsic dynamics of the protein show high centrality value.

## 3. Results

### 3.1. Sequence Space Analysis

#### 3.1.1. Coupling Analysis

**Figure 1:**
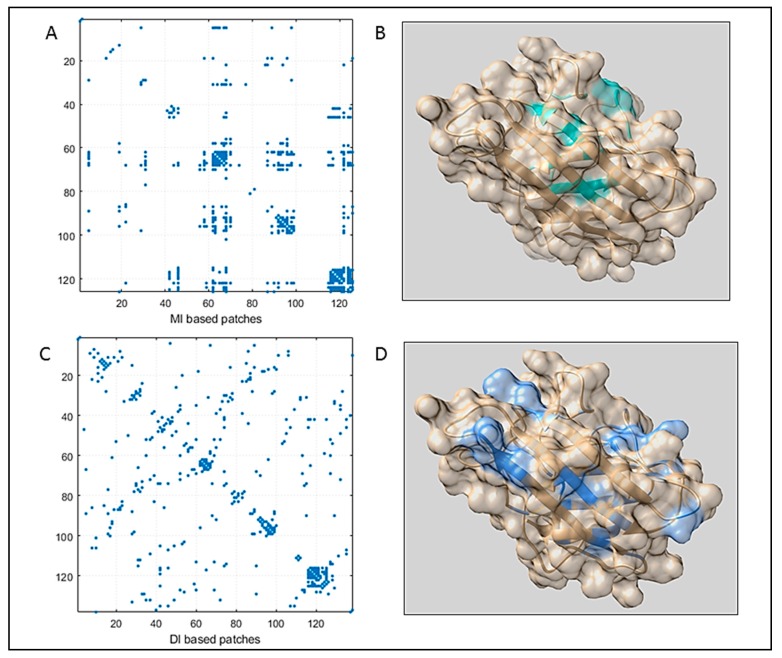
The figure displays the (**A**) Mutual Information (MI) map generated for Superoxide dismutase SOD. Here, residue pairs are clustered along the sequence. (**B**) Top MI pairs imposing contact were mapped in the consensus structure (light sea green patches). (**C**) Direct Information (DI) map for SOD, where coupled pairs were almost evenly distributed throughout the sequence. It predicted more accurately which coupled patches imposed contacts. (**D**) Top DI pairs were highlighted in the consensus structure (in cornflower blue color).

In order to explore how one position of the protein co-varies with other positions from an evolutionary perspective, DCA was implemented on the MS_align_ to retrieve highly co-varying coupled pairs. The coupling study revealed positional correlations of residues; depending upon the impact of whole sequence, we obtained MI_cij_ that corresponded to the MI scores and, by disentangling indirect interactions from the direct ones, DI_cij_ was generated that specifies the DI scores. From MI and DI scores, a probable set of interacting partners (both direct and indirect correlations for MI and direct interactions for DI) were obtained (Figure 1A,B), among which top MI and DI pairs (pairs having top MI and DI value) imposing contacts are listed in Table 2 and Table 3 respectively. Many residue pairs, being close neighbors through the sequence, were also introduced with high MI and DI scores [35]. To avert such scorings, residue pairs with a minimum separation of five positions along the sequence were considered for contact prediction. High coupling score implicates coordinated variations at specific positions, which, in turn, relates the co-variation of the amino acid residues.

The numbers of contacts to be considered from high-ranking MI pairs were lower than the DI prediction (Table 2 and Table 3). Out of the selected top MI pairs, 12 pairs were found to impose contacts (indicated by bold letters in Table 2). The DI map resulted in more accurate contact prediction. Predicted coupled residues from DI were observed to be evenly distributed throughout the sequence. Out of selected top DI pairs, 18 coupled pairs were found to impose contacts (indicated by bold letters in Table 3). Among those 36 residues, 17 residues were positioned in the loop region of the consensus structure (Supplementary Materials Figure S1). The consensus structure, generated from the consensus sequence, was a clear representation of the SOD family (a perfect sample, where we can map the evolutionary sequence space information). Hence, this structure can be implemented as a perfect bridge between the sequence space and structure space.

**Figure 2:**
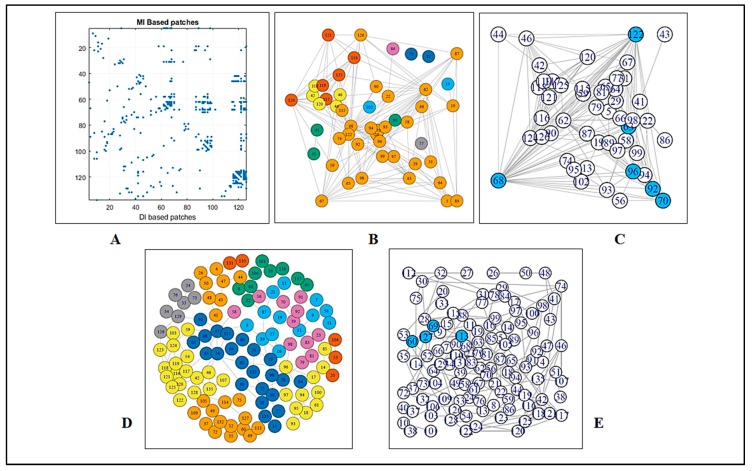
The figure displays a (**A**) contact map of MI-based residues over DI-based residues, where the distribution of the coupled pairs is compared. (**B**) An MI coupling score-based network and respective modules to define the strength of the network. (**C**) Blue nodes are signified as the members of the maximal clique from the MI-based network. (**D**) A DI coupling score-based network and respective modules to define the strength of the network. (**E**) Blue nodes are signified as the members of the maximal clique from the DI-based network.

**Figure 3:**
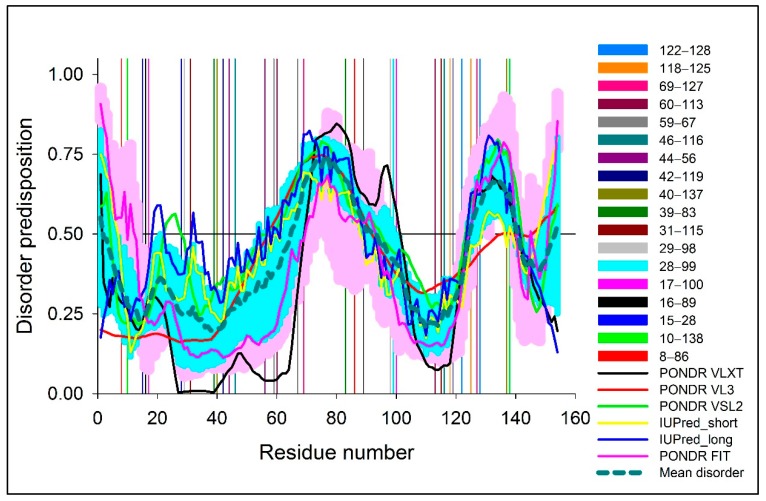
Multiparametric analysis of the intrinsic disorder predisposition of WT SOD1 evaluated by PONDR^®^ VLXT (black line), PONDR^®^ VL3 (red line), PONDR^®^ VSL2 (green line), PONDR^®^ FIT (pink line), IUPred_short (yellow line), and IUPred_long (blue line). Light pink shadow around PONDR^®^ FIT curves shows error distribution. Bold, dashed, dark cyan line shows the mean disorder propensity calculated by averaging disorder profiles of individual predictors, whereas the light cyan shadows around the corresponding curves represent the error distribution. Differently colored vertical bars reflect positions of the DI pairs. The corresponding color coding is described in the figure notes.

To detect the strongly connected local nodes, sequence-based community networks were generated (Figure 2B,D). In order to understand which statistical couplings impose contacts, residue pairs were selected by implementing the abovementioned criteria and were also mapped (Figure 1A,B). To further illustrate the positioning of the highly co-varying coupled pairs involved in direct interactions relative to the predicted intrinsically disordered regions of WT SOD1, Figure 3 represents the intrinsic disorder profile of this protein generated by a set of commonly used intrinsic disorder predictors, where locations of these co-varying coupled pairs are shown by differently colored vertical bars. The complementary information on the mean predicted disorder scores (PDSs) of high-ranking MI and DI pairs are shown in Table 2 and Table 3 correspondingly. In computational disorder analysis, residues/regions with the PDS values exceeding the threshold of 0.5 are considered as disordered, whereas the residues/regions with the PDS values between 0.2 and 0.5 are considered as flexible. This analysis revealed that all residues predicted to be involved in the co-variation are expected to be either flexible or intrinsically disordered. Table 2 shows that one coupled pair (residues 69 and 127) is intrinsically disordered, whereas in half of the DI co-varying coupled pairs, at least one of the residues is intrinsically disordered. Furthermore, in more than half of the remaining cases, at least one of the residues in the co-varying coupled pair has a PDS value exceeding 0.3. This is an important observation indicating that the observed covariance is typically not based on the intrinsic predisposition of the regions containing co-varying pairs to be ordered.

#### 3.1.2. Graph Theoretical Modelling

A fluctuation at a single critical residue can affect multiple other residues, i.e., the residue may have an enormous impact on the overall network as well as on the internal dynamics of a protein. We deployed a network model using modularity to determine the residues which have the highest impact in crafting the amino acid interaction pattern in SOD. In the case of MI, a complete subgraph was found composed of six amino acid residues (i.e., 63, 68, 70, 92, 96, and 122), among which, four were housed in the loop region (Supplementary Materials Figure S1). By considering maximal clique, in the case of DI, four residues (i.e., 60, 69, 113, and 127) were found to construct a complete subgraph (Figure 2E), i.e., substitution at any of the four residues would distort the subgraph, hence the overall network. Out of these four nodes, three were found to be housed in the loop region.

### 3.2. Structure Network Analysis

**Figure 4:**
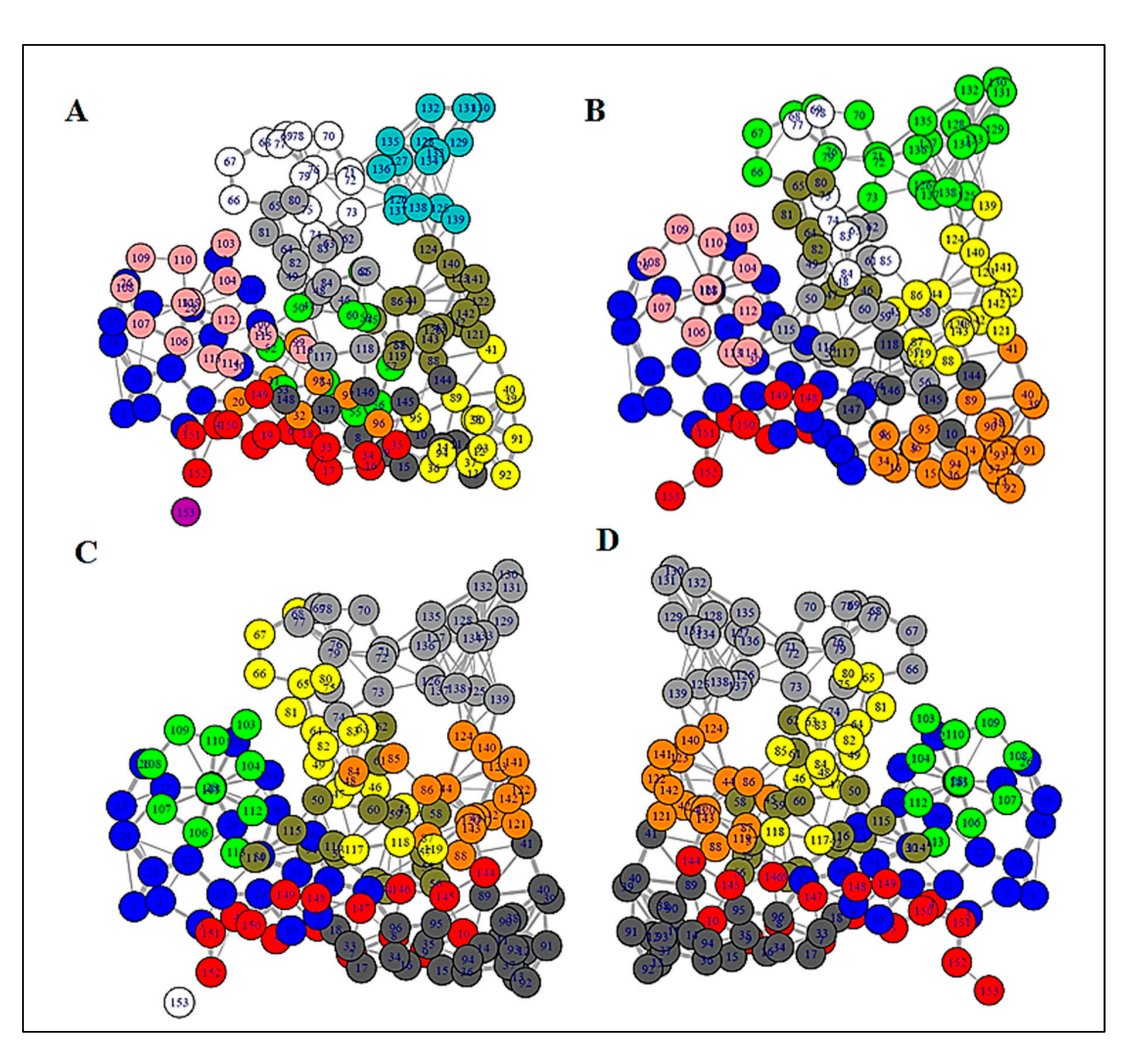
Full residue network of different variants showing inter-connection between all the residues. Residues denoted by same color code have very high connectivity and are grouped into the same clusters. (**A**) WT SOD1, (**B**) L7, (**C**) Zn-SOD1, and (**D**) apo-SOD1.

**Figure 5:**
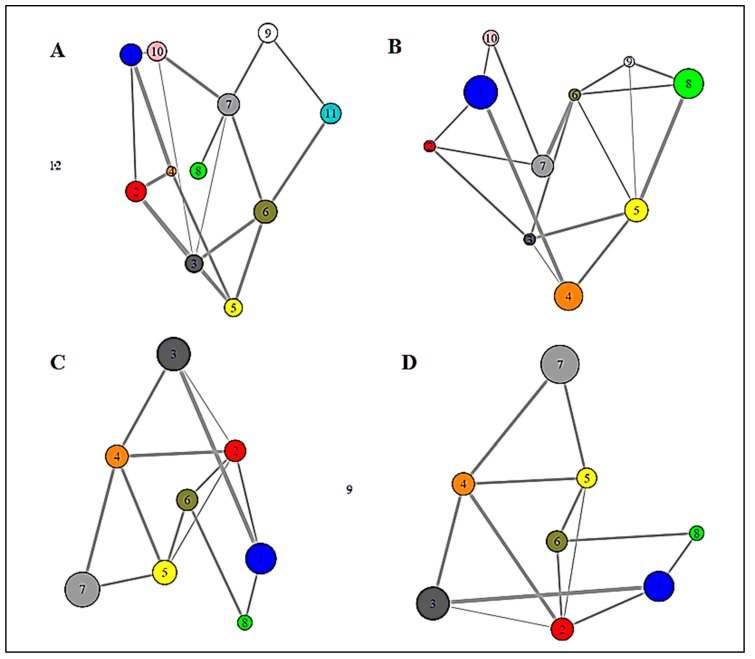
Community cluster network of different mutant models of human SOD1 showing that tightly intra-connected residues are clustered into coarse-grained communities; (**A**) in WT SOD1, all the residues are patched into 15 clusters, (**B**) L7_SOD1 has 10 communities, (**C**) Zn-SOD1 consists of 9 clusters, and (**D**) an apo mutant where both the metals are absent is formed of 8 communities.

In order to unravel the internal organization and inter-dependency of residues, structure network analysis was deployed. By generating an all residue network (Figure 4) coupled with community clustering, the evolutionarily coupled co-varying patches in WT SOD1 and its variants were mapped. In the case of WT SOD1, which contained both metal ion co-factors, Cu and Zn, the coarse-grained community network was composed of 12 coarse-grained clusters (Figure 5A). On the contrary, fully de-metallated apo SOD1 was built of only eight coarse-grained clusters (Figure 5D). In the case of partially de-metallated variants and the stretch mutants, the numbers of coarse-grained community clusters were found to be an intermediate between those of WT and apo SOD1. A community cluster network of Zn-SOD1 (Figure 5C), Cu-SOD1 (Supplementary Materials Figure S2), and L7 (Figure 5B) variants were composed of 9, 10, and 10 clusters, respectively. Strikingly, both the L4 and L4S variants with disrupted metal co-ordination sites (owing to the mutation of the specific metal-binding residues) also had 8 clusters (Supplementary Materials Figure S2), comparable with the number and clustering organization of apo SOD1. In order to unveil how the directly coupled pairs were positioned in various mutants, all residue community cluster networks of different models were analyzed. All the directly coupled pairs obtained from sequence space analysis were found to be positioned either in the same cluster or in two highly connected clusters. These results were obtained for all mutant models analyzed in this study. For WT SOD1, directly coupled residues 139 and 141 belonged to the same cluster (cluster 3; Figure 4A and Figure 5A). Whereas, in all the residue cluster networks of apo SOD1 (Figure 4D and Figure 5D) and Zn-SOD1 (Figure 4C and Figure 5C), they were housed in the two integrally connected clusters (clusters 7 and 4, respectively). In the case of the L7 stretch mutant, highly coupled residues 139 and 141 were positioned in cluster 5 (Figure 4B and Figure 5B). In this fashion, we explored how the evolutionarily coupled co-varying patches were positioned differentially depending upon metal residence as well as concomitant steric restrictions of the loops.

**Figure 6:**
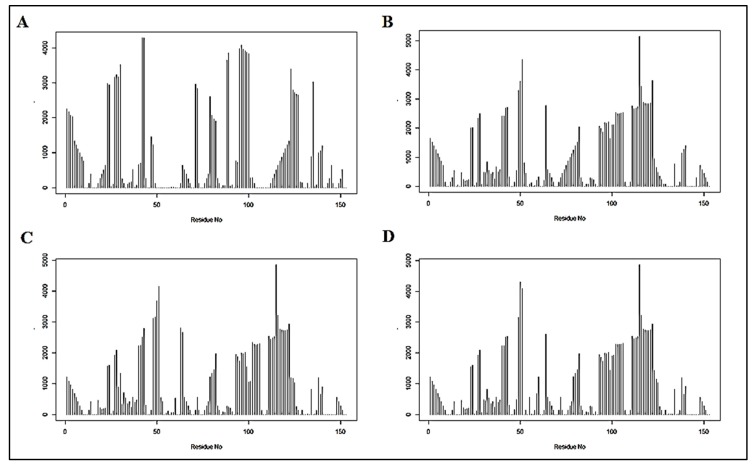
Betweenness centrality plot of different SOD1 mutants having different network distributions as well as internal dynamics; (**A**) WT protein with both the metal ion co-factors, (**B**) loop VII domain, (**C**) Cu mutant, i.e., mono-metallic Zn-SOD1, and (**D**) apo-SOD1.

By deploying betweenness centrality calculations, the influence of a particular node on the internal dynamics of different models was decoded [41]. In the case of WT SOD1, the residue stretches spanning from around 50 to 80 showed low betweenness centrality value (Figure 6A). This is consistent with the decrease in organizational fluctuation of the loop IV in the metal-bound state.

## 4. Discussion

From the MI and DI scores, probable sets of interacting partners (residues) were defined through the networks, where each node represented an amino acid residue and the edges revealed the interactions, i.e., the coupling among the residues. By exploring the top DI pairs, it was observed that most of the co-varying residues participating in coupling interactions were positioned mainly in loops IV and VII. Resorting to graph theoretical models, we could conclusively infer the importance of the loop regions in SOD. From the graph theoretical model, it becomes very apparent that, although the overall structural integrity of SOD1 is predominantly determined by its β-sheet structure, the unstructured loop segments also have a key contribution. Interestingly, many of these coupled co-varying residues have already been linked with SOD1 aggregation and ALS from the structural and physiological perspectives. A mutation in position 8 has been reported to have reduced enzymatic activity and has been isolated from ALS patients [43]. There are reports of an H46R mutation in the Cu/Zn SOD gene which has been highly related to an unique subtype of familial ALS [44] and non-native conformational changes leading to a gain of interaction among dimers further propagating to higher-order arrays [45]. Further, there are reports which state a mutation at position 58 heavily impacts on Cu loading, owing to the impaired chaperone interaction [46] and promotes fibrillar aggregate formation [47]. It is interesting to note that residues around position 58, also deciphered in our co-evolution analysis, have been reported to be extremely critical in deciding the dimerization propensity, as the stretch involves residue associated with intra-subunit disulfide bond and an increased loop flexibility [48]. Mutation at residue 68 has also been reported in clinical cases of ALS [49]. Mutations at positions 118 and 125 have also been reported as novel exonic mutations in clinical cases of ALS [50].

We performed structure network analysis to understand how global organization in SOD1 is dictated by loops IV and VII which, in turn, house the majority of these evolutionarily co-evolving residues. The apo form of SOD1 with an absence of metal-ion co-factors represents a completely opposite state compared to WT SOD1. Since SOD1 is complex system with heterogeneous secondary structural organizations and co-factor ions orchestrating the structural fine tunings, pathogenic forms of SOD1 show wide disparities in terms of protein stabilities. But most ALS-associated mutations have been reported to have the greatest impact on the immature form of SOD1 with destabilized metal free states [51]. Betweenness centrality profiles in our study revealed that the metal pockets (i.e., Cu and Zn co-ordination sites) in the presence of metal ion co-factors exhibit structural rigidity. For other mutants, centrality values for this same stretch were found to be higher than the WT (Figure 6). Interestingly, since all the other variants had disrupted metal co-ordination, they showed near equal centrality values for the aforementioned stretch. Difference in the centrality value between the WT protein and other variants strongly indicates the importance, as well as the influence, of those sites on the intrinsic dynamics of the protein. The apo and Zn-SOD1 shared almost a similar range of centrality throughout intrinsically disordered domains I and II (IDD I and IDD II). We provide a clear picture of how the internal dynamics of SOD1 gradually changes upon metal co-ordination. This can be directly correlated with the structural stability of SOD1 earlier reported. Earlier reports have stated how Cu and Zn co-ordinations stand extremely important in the context of structural integrity and preventing aggregation by, respectively, stabilizing the intra-subunit disulfide linkage and promoting the folding in the two disorder loops and, hence, creating the catalytic subunit and concomitant stabilization of the global structure [23,51].

**Figure 7:**
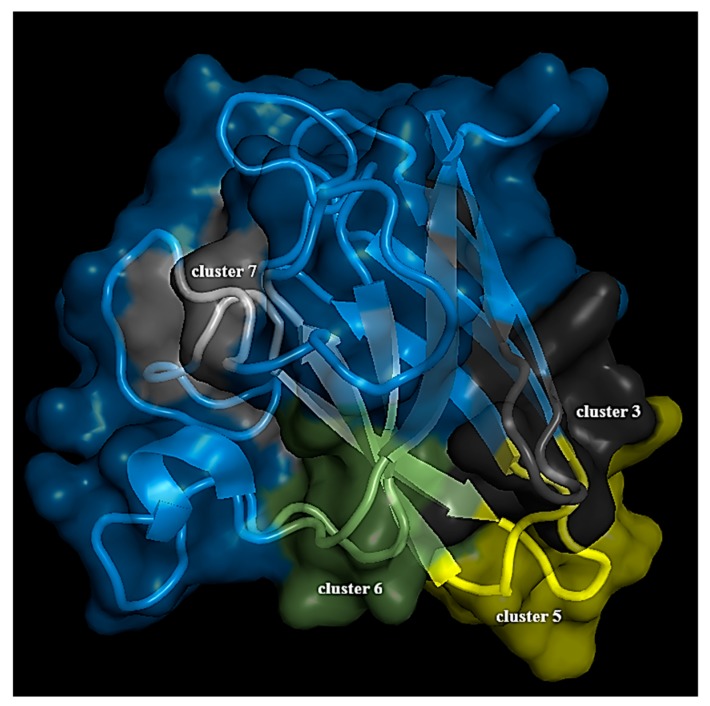
The WT SOD1 protein. Community clusters composed of important residues are highlighted.

The change in the number of community clusters, as observed in our structure network analysis, from de-metallated to fully metallated states through the intermediate partially metallated states and other variants unraveled that the metal co-ordination sites, and their micro-environments are tightly constrained. The increment in the number of clusters in SOD1 under completely metallated conditions indicates smaller local arrangements resulting from Cu and Zn micro-environments (Figure 5A). Here, the fluctuation in the residues near the metal co-ordination sites almost diminishes, having insignificant contribution to internal dynamics, evidenced by a negligible betweenness centrality value (Figure 6A). The absence of metal ions disrupts these smaller local arrangements with a concomitant impact on the internal dynamics emanating from the residues making up the loop regions of SOD1, which otherwise crafts the metal micro-environments. Thus, the number of clusters decreases to eight in the case of apo SOD1 (Figure 5D). By considering all these facts, residues in cluster 3, 5, 6, and 7 in the community cluster network of the WT protein were considered to be very much significant (Figure 7).

In the absence of metal ions, either in the partial mono-metallated state or complete de-metallated state, the extended loops, due to the fact of their intrinsically disordered nature, support a continuum of conformational states and transitions. The binding of metal ion co-factors to an intrinsically unstructured protein complements disorder to order transition that is concomitant to an entropic cost [52]. Here, thermodynamic stability is guided by favorable enthalpy contribution, which represents the enthalpy–entropy compensation [52]. These are all internal events and remain synchronized with the metal co-ordination in SOD1. This renders a cryptic disorder in proteins like SOD1, where the metal ion cofactors upon entry conceals the local disorder and locks the loop region in its state of restricted mobility. Our evolutionary analysis pinpoints specific residues which are co-evolving and are hence extremely critical for SOD1’s biological relevance. Interestingly, mutations associated with many of these positions have already been associated to ALS. Further, our analysis reveals some novel sites which have not been associated with ALS earlier and yet are critical and, hence, co-evolving.

## 5. Conclusions

Our study provides a holistic view of the SOD1 structure encompassing the evolutionary details and the structure network maps and chalks out the gradual transitions which happen on SOD1’s transition from its de-metallated apo state to bi-metallated Cu and Zn bound states via the mono-metallated stages. The evolutionary analysis presented in this work decodes specific positions that are evolutionarily co-varying and are extremely critical in terms of the stability of the protein structure. We identify and validate novel stretches in SOD1 which have earlier not been associated with SOD1 instability. Furthermore, our analysis reveals residue-specific properties in terms of their pairwise interactions and contribution towards the internal dynamics as well as the changes in these traits under the alteration of metal co-ordination contexts.

## Figures and Tables

**Table 1:** Substituted residues.

Mutant Name	Mutations
apo-SOD1	H63F
Cu-SOD1	H120F
Zn-SOD1	H71F
L4_SOD1	N65S, L67P, G72S, D76Y, H80A
L7_SOD1	D124G, D125H, L126S, S134N, N139K, L144F
L4S_SOD1	G72S, D76Y, H80A, L84F, A89T, D90A, G93C, A95G

**Table 2:** Top MI Pairs with mean Predicted Disorder Score (PDS) considering the standard deviation.

Residue Number (Mean PDS)	Coupling Pairs (Mean PDS)	MI Score
**29 (0.25 ± 0.16)**	**98 (0.43 ± 0.11)**	0.64260
**42 (0.21 ± 0.10)**	**117 (0.24 ± 0.09)**	0.703328
**44 (0.26 ± 0.13)**	**122 (0.36 ± 0.05)**	0.593079
**46 (0.27 ± 0.11)**	**115 (0.23 ± 0.09)**	0.577762
58 (0.38 ± 0.18)	68 (0.60 ± 0.12)	0.615481
62 (0.44 ± 0.19)	116 (0.26 ± 0.10)	0.583716
**67 (0.56 ± 0.14)**	**124 (0.48 ± 0.06)**	0.608395
**70 (0.68 ± 0.10)**	**96 (0.47 ± 0.11)**	0.603931
99 (0.42 ± 0.10)	122 (0.36 ± 0.05)	0.603343
118 (0.27 ± 0.09)	125 (0.52 ± 0.07)	0.6989

The most important pairs are indicated in Table 2 by bold face.

**Table 3:** Top DI Pairs with mean PDS scores considering the standard deviation.

Residue Number (Mean PDS)	Coupling Pairs (Mean PDS)	DI Score
8 (0.33 ± 0.12)	86 (0.59 ± 0.05)	0.108738
**10 (0.28 ± 0.09)**	**138 (0.63 ± 0.09)**	0.122847
**15 (0.24 ± 0.05)**	**28 (0.25 ± 0.16)**	0.125331
16 (0.25 ± 0.05)	89 (0.54 ± 0.04)	0.109716
17 (0.27 ± 0.07)	100 (0.41 ± 0.09)	0.0900074
28 (0.25 ± 0.16)	99 (0.42 ± 0.10)	0.131024
29 (0.25 ± 0.16)	98 (0.43 ± 0.11)	0.095733
31 (0.25 ± 0.16)	115 (0.23 ± 0.09)	0.0851411
**39 (0.21 ± 0.11)**	**83 (0.67 ± 0.09)**	0.0948818
**40 (0.21 ± 0.11)**	**137 (0.62 ± 0.12)**	0.249519
**42 (0.21 ± 0.10)**	**119 (0.28 ± 0.07)**	0.105276
44 (0.26 ± 0.13)	56 (0.34 ± 0.17)	0.0880899
**46 (0.27 ± 0.11)**	**116 (0.26 ± 0.10)**	0.0962272
59 (0.38 ± 0.19)	67 (0.56 ± 0.14)	0.107917
**60 (0.39 ± 0.19)**	**113 (0.22 ± 0.08)**	0.156108
**69 (0.65 ± 0.13)**	**127 (0.58 ± 0.09)**	0.126032
118 (0.27 ± 0.09)	125 (0.52 ± 0.07)	0.100409
**122 (0.36 ± 0.05)**	**128 (0.58 ± 0.09)**	0.156898

The most important pairs are indicated in Table 2 by bold face.

## Supplementary Materials

The following are available online at https://www.mdpi.com/2218-273X/9/12/826/s1, Figure S1: This figure designates the consensus structure. (A) Spheres highlighted by magenta color indicates the residues from top MI pairs positioned in loop region. (B) Spheres emphasized by deep blue color designates the residues from top DI pair that were housed in the loop region, Figure S2: (A and D) All residue network and community cluster network of Cu-SOD1. (B and E) All residue network and community cluster network of L4_SOD1. (C and F) Full residue network and simplified community network of L4S_SOD1, Figure S3: Betweenness centrality profile of (A) Cu-SOD1, (B) L4_SOD1 and (C) L4S_SOD1, Table S1: WT SOD1 cluster members, Table S2: apo-SOD1 cluster members, Table S3: Zn SOD1 cluster members, Table S4: Cu SOD1 cluster members, Table S5: L4 SOD1 cluster members, Table S6: L7 SOD1 cluster members, Table S7: L4S SOD1 cluster members, Table S8: DI network members, Table S9: MI network members.

## Abbreviation

SOD1: human Cu, Zn Superoxide Dismutase; ALS: Amyotrophic Lateral Sclerosis; MSA: Multiple Sequence Alignment; HMM: Hidden Markov Model; PDS: Predicted Disorder Score DCA: Direct Coupling Analysis; MI: Mutual Information; DI: Direct Information; NMA: Normal Mode Analysis; IDD: Intrinsically Disordered Protein.
