# Supplementary material for: Evolutionary Analyses of Sequence and Structure Space Unravel the Structural Facets of SOD1
Source: Biomolecules. 2019 Dec 4;9(12):826. doi: 10.3390/biom9120826 (PMC6995586; doi:10.3390/biom9120826)
Supplement: Supplementary file 1 [file biomolecules-09-00826-s001.pdf]

Evolutionary analyses of sequence and structure space unravel the structural facets of SOD1

Sourav Chowdhury [1,2,†], Dwipanjan Sanyal [1,†], Sagnik Sen [3,†], Vladimir N. Uversky,[4,5], Ujjwal Maulik [3] and Krishnananda Chattopadhyay [1,*]

1. Protein Folding and Dynamics Group, Structural Biology and Bio-informatics Division, CSIR-Indian Institute of Chemical Biology,4 Raja S.C.Mullick Road, Kolkata 700032, India; dwipanjan92@gmail.com
2. Chemistry and Chemical Biology, Harvard University, 12 Oxford Street, Cambridge, MA 02138, USA; sourav_chowdhury@fas.harvard.edu
3. Department of Computer Science, Jadavpur University, Kolkata 700032, India; sagnik.sen2008@gmail.com (S.S.); umaulik@cse.jdvu.ac.in (U.M.)
4. Department of Molecular Medicine and USF Health Byrd Alzheimer's Research Institute, Morsani College of Medicine, University of South Florida, 12901 Bruce B. Downs Blvd. MDC07, Tampa, FL 33612, USA; vuversky@health.usf.edu
5. Laboratory of New Methods in Biology, Institute for Biological Instrumentation, Russian Academy of Sciences, Pushchino 142290, Moscow Region, Russia

† Contributed Equally

* Correspondence: krish@iicb.res.in; Tel.: +91 33 2499584

Received: 10 October 2019; Accepted: 16 November 2019; Published: date

Supplementary Figures

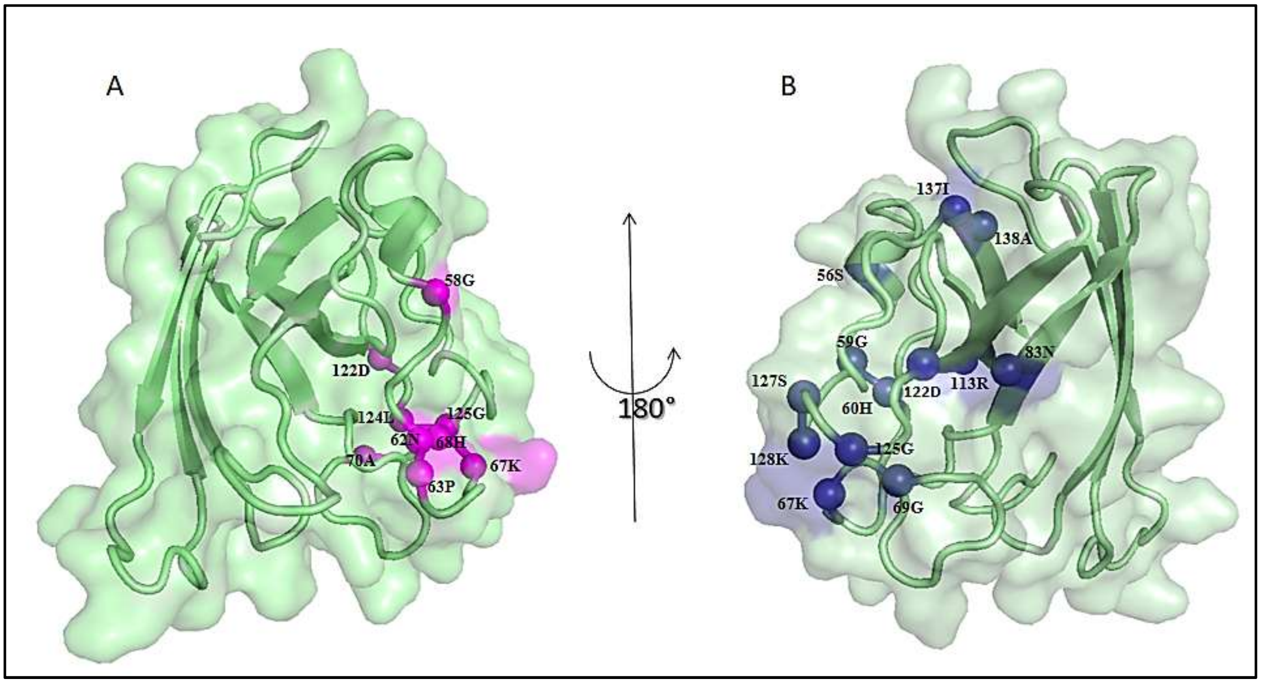

Supplementary Figure 1. This figure designates the consensus structure. (A) Spheres highlighted by magenta color indicates the residues from top MI pairs positioned in loop region. (B) Spheres emphasized by deep blue color designates the residues from top DI pair that were housed in the loop region.

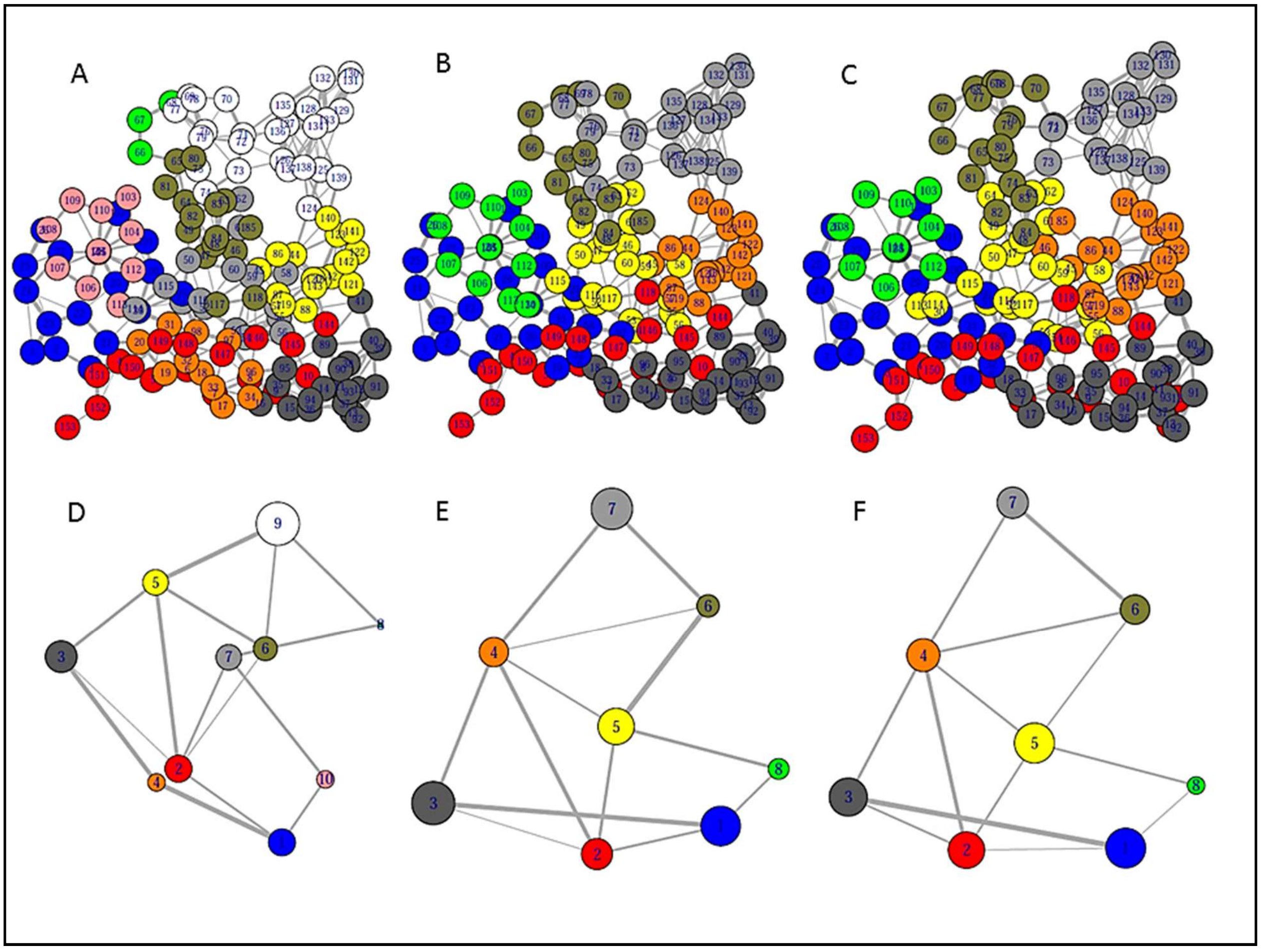

Supplementary Figure 2. (A and D) All residue network and community cluster network of Cu-SOD1. (B and E) All residue network and community cluster network of L4_SOD1. (C and F) Full residue network and simplified community network of L4S_SOD1.

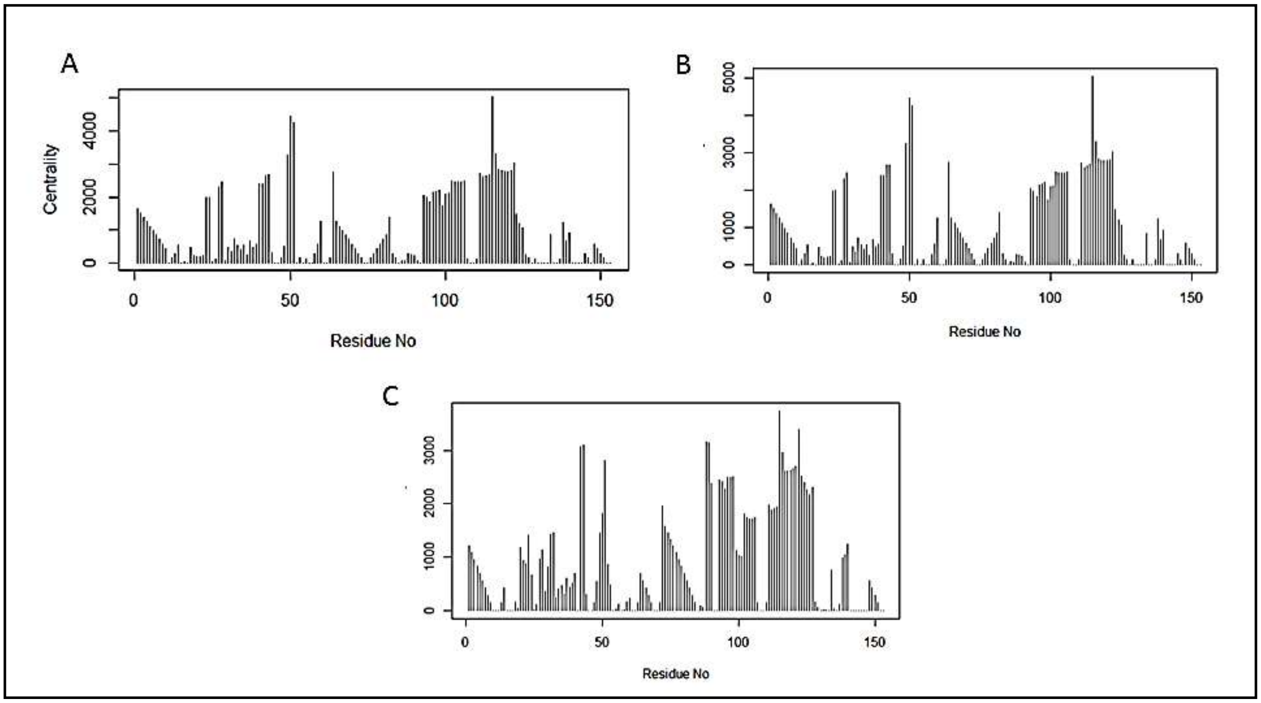

Supplementary Figure 3. Betweenness centrality profile of (A) Cu-SOD1, (B) L4_SOD1 and (C) L4S_SOD1.

Supplementary Table 1: WT SOD1 cluster members

Cluster id	Size	Residue members
1	16	c(1:3, 21:30, 100:102)
2	15	c(4:7, 16:19, 33:35, 149:152)
**3**	13	c(8:15, 144:148)
4	7	c(20, 31:32, 96:99)
**5**	13	c(36:41, 89:95)
**6**	17	c(42:45, 86:88, 119:124, 140:143)
**7**	16	c(46:49, 62:65, 80:85, 117:118)
8	12	50:61
9	14	66:79
10	14	103:116
11	15	125:139
12	1	153

Important clusters are highlighted in bold face

Supplementary Table 2: apo-SOD1 cluster members

Cluster id	Size	Members
1	23	c(1:3, 19:32, 97:102)
2	17	c(4:10, 144:153)
3	25	c(11:18, 33:41, 89:96)
4	17	c(42:45, 86:88, 119:124, 140:143)
5	15	c(46:49, 63:65, 80:85, 117:118)

6	16	c(50:62, 114:116)
7	29	c(66:79, 125:139)
8	11	103:113

Supplementary Table 3: Zn SOD1 cluster members

Cluster id	Size	Members
1	23	c(1:3, 19:32, 97:102)
2	16	c(4:10, 144:152)
3	25	c(11:18, 33:41, 89:96)
4	17	c(42:44, 84:88, 120:124, 140:143)
5	18	c(45:49, 63:68, 80:83, 117:119)
6	16	c(50:62, 114:116)
7	26	c(69:79, 125:139)
8	11	103:113
9	1	153

Supplementary Table 4: Cu SOD1 cluster members

Cluster id	Size	Members
1	17	c(1:3, 21:30, 99:102)
2	17	c(4:10, 144:153)
3	20	c(11:16, 35:41, 89:95)
4	11	c(17:20, 31:34, 96:98)
5	16	c(42:45, 86:88, 119:123, 140:143)
6	15	c(46:49, 63:65, 80:85, 117:118)
7	16	c(50:62, 114:116)
8	3	66:68
9	27	c(69:79, 124:139)
10	11	103:113

Supplementary Table 5: L4 SOD1 cluster members

Cluster id	Size	Members
1	23	c(1:3, 19:32, 97:102)
2	18	c(4:10, 118, 144:153)
3	25	c(11:18, 33:41, 89:96)
4	17	c(42:45, 86:88, 119:124, 140:143)
5	21	c(46:63, 115:117)
6	13	c(64:70, 80:85)
7	24	c(71:79, 125:139)
8	12	103:114

Supplementary Table 6: L7 SOD1 cluster members

Cluster id	Size	Members
1	26	c(1:3, 17:33, 97:102)
2	9	c(4:6, 148:153)
3	9	c(7:10, 118, 144:147)
4	22	c(11:16, 34:41, 89:96)
5	18	c(42:45, 86:88, 119:124, 139:143)
6	9	c(46:48, 64:65, 80:82, 117)
7	17	c(49:63, 115:116)
8	23	c(66:73, 79, 125:138)
9	8	c(74:78, 83:85)
10	12	103:114

Supplementary Table 7: L4S SOD1 cluster members

Cluster id	Size	Members
1	23	c(1:3, 19:32, 97:102)
2	21	c(4:13, 118, 144:153)
3	22	c(14:18, 33:41, 89:96)
4	19	c(42:46, 85:88, 119:124, 140:143)
5	23	c(47:64, 113:117)
6	17	c(65:70, 74:84)
7	18	c(71:73, 125:139)
8	10	103:112

Supplementary Table 8: DI network members

Color	Size	Members
Yellow	27	c(14,17,18,42,46,54,49,61,93:97,100,103,107,117:125,120,135)
Blue	22	c(15,27:32,62:68,74,78,84,88,90,112,115,133)
Green	9	c(8,10,22,40,86,101,106,137,138)
Grey	7	c(24,33,34,73,76,126,129)
Violet	11	c(23,39,56,58,70,79,81,83,91,92,98)
Brown	3	c(20,53,104)
Turquoise	13	c(5,7,9,11,13,16,19,21,38,51,77,87,89)
Apricot	8	c(4,26,41,43,44,47,48,50)
Tangerine	14	c(36,37,49,52,60,69,72,79,105,109,113,114,121,132)

Supplementary Table 9: MI network members

Color	Number of Members	Members
Violet	1	86
Blue	2	79,81
Grey	1	77
Turquoise	2	13,102
Green	3	c(95,41,43)
Yellow	5	c(42,44,46,118,120)
Brown	6	c(116,117,119,121,124,125)
Tangerine	24	c(5,19,22,29,31,56,58,59,63:70,89,92:94,99,97,122)
